# Human endothelial cell-derived exosomal microRNA-99a/b drives a sustained inflammatory response during sepsis by inhibiting mTOR expression

**DOI:** 10.3389/fcimb.2022.854126

**Published:** 2022-08-18

**Authors:** Glenn Fitzpatrick, Danielle Nader, Rebecca Watkin, Claire E. McCoy, Gerard F. Curley, Steven W. Kerrigan

**Affiliations:** ^1^ Cardiovascular Infection Research Group, Dublin, Ireland; ^2^ School of Pharmacy and Biomolecular Sciences, RCSI University of Medicine and Health Sciences, Dublin, Ireland; ^3^ Department of Anaesthesia and Critical Care Medicine, RCSI University of Medicine and Health Sciences, Beaumont Hospital, Dublin, Ireland

**Keywords:** sepsis, inflammation, exosomes, endothelial cell, mTOR, microRNA, infection

## Abstract

The pathophysiology of sepsis and its accompanying hyper-inflammatory response are key events that lead to multi-organ failure and death. A growing body of literature now suggests that the vascular endothelium plays a critical role in driving early events of sepsis progression. In this study, we demonstrate how endothelial-derived exosomes contribute to a successive pro-inflammatory phenotype of monocytes. Exosomes isolated from *S. aureus* infected endothelial cells drive both CD11b and MHCII expression in monocytes and contribute dysregulated cytokine production. Conversely, healthy endothelial exosomes had no major effect. microRNA (miRNA) profiling of exosomes identified miR-99 upregulation which we hypothesised as driving this phenotypic change through mechanistic target of rapamycin (mTOR). Knockdown of mTOR with miR-99a and miR-99b mimetics in *S. aureus* infected monocytes increased IL-6 and decreased IL-10 production. Interestingly, inhibition of miRNAs with antagomirs has the opposing effect. Collectively, endothelial exosomes are driving a pro-inflammatory phenotype in monocytes through dysregulated expression of miR-99a and miR-99b.

## Introduction

Sepsis is a multifaceted disorder caused by a sustained and often excessive dysregulated response by the host to an infectious nidus that results in acute organ dysfunction and carries a high risk of mortality. In 2017, 48.9 million incidence cases of sepsis were reported with a mortality of 11 million people accounting for 19.8% of all global deaths (Rudd et al., 2020) and as such the World Health Assembly and WHO made sepsis a global health priority. Despite significant advances in our understanding of both early and late stage pathogenesis, new interventions to treat sepsis still remains elusive. Current treatment options are limited and rely on early recognition, resuscitation, source control and antibiotic therapy.

The innate immune response is the first line of defence and is responsible for responding to invading pathogens in an appropriate manner. Sepsis develops when the normal innate immune response to an infection becomes amplified and subsequently dysregulated. This results in an excessive release of cytokines, chemokines and other inflammatory mediators leading to an uncontrolled cytokine storm. Typically, cytokines regulate inflammatory responses by controlling migration of immune cells to the focus of infection. This is a critical step that contains the infection and prevents it from becoming a more serious systemic infection. A dysregulated cytokine release triggers endothelial dysfunction, characterized by vasodilation and increased capillary permeability which results in hypotension, a decrease in plasma concentration due to macromolecular extravasation and oedema. The increase in permeability facilitates dissemination of the infection from the focal point in the systemic circulation and causes further disturbance to regulatory mechanisms causing remote organ dysfunction and eventual failure. While it is clear the early steps in the innate immune response during sepsis can lead to a cytokine storm it is often followed by a prolonged state of immunosuppression. This immunoparalysis causes an impairment of both the innate and adaptive immune response which leads to further tissue damage, multiple organ failure, and death.

Currently the mechanisms that drive the early stages of the immune response that leads to the cytokine storm is poorly understood. A growing body of literature now suggest that the vascular endothelium may be playing an important role in sepsis progression through a dysregulated miRNome (Chu et al., 2016). MiRNA’s constitute a family of endogenously expressed non-coding RNA, approx. 18 – 22 nucleotides, that acts as post-transcriptional repressors of gene expression. Both 3’ and 5’ miRNA transcripts suppress gene expression through interactions with the 3’ UTR, 5’ UTR, and CDS of mRNA. Interestingly, miRNA’s can be found extracellularly in bodily fluids such as plasma, serum, saliva, and urine (Felekkis et al., 2010; Cui et al., 2019). These circulating miRNAs are protected from the enzymatic activity of RNases by being tethered to Argonaute (AGO) proteins (Buscaglia and Li, 2011; Cortez et al., 2011; Hatziapostolou et al., 2013; Soliman et al., 2018; Cui et al., 2019; Salvi et al., 2019). Alternatively, miRNA’s can be packaged into plasma membrane lipid vesicles including apoptotic bodies, microparticles and exosomes. Exosomes are lipid nanovesicles which act as transport (30-100nm) and form through the inward invagination of multivesicular bodies. They are subsequently released upon fusion with the plasma membrane. Initially it was believed that exosomes were nothing more than cellular waste, however accumulating evidence in the literature now suggest that they play a vital role in numerous physiological and pathological processes and influence the function of various cell types (Samanta et al., 2018).

As the vascular endothelial cells are among the first cells in the body that come into contact with invading pathogens, the delivery of dysregulated miRNome by endothelial cells encapsulated in exosomes may be driving the host’s response to infection in sepsis. In this study, we demonstrated that *S. aureus* infection results in the release of endothelial cell derived exosomes that contain a critical miRNA, miR99a/b that when taken up by immune cells is capable of driving an uncontrolled release of pro-inflammatory cytokines by targeting the mechanistic target of rapamycin (mTOR), a key protein involved in regulating the immune response.

## Materials and methods

All reagents used for experimentation were purchased from Thermo Fischer (Dublin, Ireland) unless otherwise stated.

### Blood collection & plasma preparation

Whole blood was obtained from healthy donors and anticoagulated using 200 ATU/mL Heparin (Rafludin, Celegene, UK). Platelet poor plasma (PPP) was isolated as previously described (Tilley et al., 2013). Approval for the collection of whole blood was obtained from the Ethics Committee in RCSI (REC 1121). Informed consent was provided in accordance with the Declaration of Helsinki. In the absence of healthy donors, pooled human PDP (Cambridge Biosciences, London, UK) was used for experimentation.

### Bacterial growth conditions

*Staphylococcus aureus* Newman Wildtype NCTC 8178 (Duthie and Lorenz, 1952) was cultured anaerobically overnight at 37°C in Brain Heart Infusion (BHI) broth as outlined previously (Kerrigan et al., 2008). Cultures were centrifuged at 3800 x g for 7 minutes, washed in PBS and adjusted to an OD_600nm_ = 1.0A.

### Human endothelial cell infection model

Human aortic endothelial cells (Promocell, Germany) were sheared at 10 dyn/cm^2^ for 24 hours in Endothelial Cell Growth Media MV (Cruinn, Wexford, Ireland). Spent media was aspirated and replaced with fresh EMV media containing 10ng/mL of TNFα for 4 hours, followed by a 1-hour incubation with platelet poor plasma (PPP). Wells were subsequently blocked with 1% Bovine Serum Albumin (BSA) in EMV media for 1 hour. Plates were infected with *Staphylococcus aureus* Newman Wildtype for 24 hours. Negative control plates received fresh media after BSA blocking and both groups were incubated for 24 hours at 37°C, 5% CO_2_. A multiplicity of infection (MOI) of 10 was used to infect HAoECs for 24 hours under static conditions.

### Endothelial cell exosome isolation

Exosomes were isolated from healthy and infected sheared human endothelial cell supernatants. Briefly, supernatants were centrifuged at 300 x g for 5 minutes to remove cells, 2500 x g for 10 minutes to remove dead cells and debris, and 3800 x g for 7 minutes to remove bacteria. Supernatants were transferred to an ultracentrifuge tube and centrifuged at 20,000 x g for 90 minutes to remove microparticles and larger vesicles. Supernatants were passed through a 0.2µm filter to ensure filtrate was free of *Staphylococcus aureus*. The filtrate was transferred to a new ultracentrifuge tube and centrifuged at 120,000 x g for 120 minutes.

### Characterisation of endothelial cell exosomes

For characterisation by Western Blot, exosomes were resuspended in RIPA buffer and passed through a 0.45 µM filter. Samples were sonicated at 15 A for 3 seconds and BCA assays were performed to determine protein concentration. Protein expression was determined using 1:500 rabbit anti-Alix IgG primary antibody (Clone: A302-938A, Bethyl laboratories), 1:200 rabbit anti-CD63 (Clone: MX-49.129.5, Santa Cruz Biotech), 1:1000 rabbit anti-Flotillin1 (Clone: AB_941621, Abcam), and 1:500 mouse anti-Tsg101 (Clone: 4A10, Genetex).

Exosomes were studied by flow cytometry using CD63+ (Clone TEA3/18) superparamagnetic capture beads (Abbexa, Cambridge, UK). Briefly, superparamagnetic capture beads were added to exosomes in 1X assay buffer and incubated in the dark overnight at room temperature. Next, a biotin primary antibody was added to each tube and incubated for 60 minutes at 4°C. The beads and exosome mixture were washed and placed on a magnetic rack for 5 minutes. Secondary antibody (Streptavidin-Phycoerythrin) was added for 30 minutes at 4°C, and samples were analysed using the Attune NxT Flow Cytometer.

To visualise exosomes, transmission electron microscopy (TEM) was performed. Superparamagnetic capture beads were incubated with exosomes in the dark overnight at room temperature. Following this, the exosome-bead mixture was placed on PELCO^®^ 200 Mesh Special Metal Grids (Ted Pella Inc, California, USA) and allowed to dry. Samples were visualised using a Hitachi Transmission Electron Microscope at 150,000X.

### Exosome staining

Human endothelial cells were cultured as previously described and seeded onto glass cover slips adhered to 6-well plates. Confluent endothelial cells were incubated with 5µM of a Near Infrared BF2-azadipyrromethene (NIR-AZA) fluorescent probe for 2 hours. Endothelial cells were fixed with 4% paraformaldehyde (Sigma) for 10 minutes before being washed three times with ice cold PBS (Sigma). Coverslips were mounted with 4′,6-diamidino-2-phenylindole [(DAPI) Sigma] and analysed using the Zeiss AxioObserver Z1 Microscope.

### MiRNA profiling in human endothelial cells and THP-1 monocytes

Exosomal-RNA (exo-RNA) from healthy and *S. aureus* infected human endothelial cells was isolated using Total Exosomes & Protein Isolation Kit as per manufacturer’s specifications. Small RNA composition (20–200 nucleotides) of exo-RNA was assessed using an AATI fragment analyser. RNA was reverse transcribed to complementary DNA (cDNA) and the expression of 384 miRNA were assessed using TaqMan^®^ Human Microarray A cards & RT-PCR for healthy and *S. aureus* infected samples. Individual qPCR assays were performed to confirm the expression of select miRNA from the TaqMan^®^ Human Microarray A cards. For individual assays, data was normalised to a semi-synthetic oligonucleotide cel-miR-39-3p (UCACCGGGUGUAAAUCAGCUUG) and both TaqMan^®^ Human Microarray A cards and individual miRNA expression data was calculated using the comparative cycle threshold (2^-ΔΔCt^) method.

Total RNA was isolated from THP-1 monocytes using the mirVana™ isolation kit as per manufacturers specifications. RNA was quantified using a NanoDrop spectrometer. Semi-synthetic miR-99a (rArArCrCrCrGrUrArGrArUrCrCrGrArUrCrUrUrGrUrG) and miR-99b (rArArCrCrCrGrUrArGrArUrCrCrGrArUrCrUrUrGrUrG) oligonucleotides (Integrated DNA Technologies, Leuven, Belgium) were used to generate standard curves (2 – 8200 fM) for absolute quantification of miR-99a and miR-99b levels. RNA was reverse transcribed to cDNA and RT-qPCR was performed as described above with data normalised to RNU48 and total miRNA concentrations determined from standard curves.

### Exosome activation of monocytes

Monocytes (10^6^ cells/well) were cultured with exosomes isolated from healthy and *S. aureus* infected endothelial cells for 24hrs. The suspension was centrifuged, washed, and resuspended in human FcR binding inhibitor (eBiosciences, UK). Monocytes were incubated with CD11b Monoclonal Antibody (M1/70), APC, and MHC Class II (I-A/I-E) Monoclonal Antibody (M5/114.15.2), PE, or isotype control. Expression of these activation markers was analysed by flow cytometry. For delivery of exosomes to monocytes, pellets were resuspended in RPMI media 1640+ GlutaMAX and incubated with monocytes for 24 and 120 hours

### Cytokine production following Staphylococcus aureus binding

Human IL-6 (240 µg/mL) and IL-10 (180 µg/mL) capture antibodies were adhered to 96 well plates overnight and subsequently blocked with a 1% BSA solution for 1 hour. Monocytes were co-cultured with healthy and *S. aureus* infected endothelial cells, and the supernatant was added to the plates for 2 hours at room temperature. IL-6 (120 ng/mL) and IL-10 (90 ng/mL) detection antibodies were added to the plates for 2 hours at room temperature followed by 0.25 mg/mL of Streptavidin-HRP for 20 minutes. Plates were washed between each step with 0.05% tween-20 in PBS. Plates were treated with an equal mixture of H_2_O_2_/tetramethylbenzidine, and the reaction was stopped after 20 minutes with 2N H_2_SO_4_. The absorbance at 450/570nm was determined in a 1420 Victor V3, Perkin Elmer plate reader, and standard curves were generated to determine unknown IL-6 and IL-10 sample values.

### Transfection of THP-1 monocytes

THP-1 monocytes (ATCC^®^ TIB-202™) were cultured in RPMI media 1640+ GlutaMAX supplemented with 10% Foetal bovine serum (FBS) and penicillin/streptomycin. Monocytes were seeded onto 6-well plates at a concentration of 1x10^6^ cells/well and a transfection mixture containing OptiMEMTM (Biosciences), Lipofectamine 3000, and miRNA mimetics and antagomirs (miR-99a, miR-99b, and negative control scrambled RNA) was added for 48 hours. Monocytes were subsequently infected with *S. aureus* for 24 hours at an MOI of 1. Following infection, cell culture supernatants containing monocytes were harvested by centrifugation at 1500 x g for 5 minutes and washed with ice cold PBS. Cleared lysates were separated on a 10% SDS-PAGE gel. Proteins were electroblotted onto PVDF membranes (Roche) for 1 h. Membranes were probed using a 1:1000 monoclonal rabbit anti-mTOR antibody (Clone: 7C10, Cell Signalling Technologies, Dublin, Ireland). Cytokine production was monitored from supernatants for IL-6 and IL-10 as previously outlined.

### Statistical analysis

Data are presented as mean ± standard error of the mean. Experiments were carried out in triplicate with a minimum of three independent experiments. Statistical differences between groups were assessed by ANOVA with Tukey *post hoc* tests or t-tests, as indicated. P-value < 0.05 was considered to be significant.

## Results

### Characterisation of human endothelial-derived exosomes

**Figure 1:**
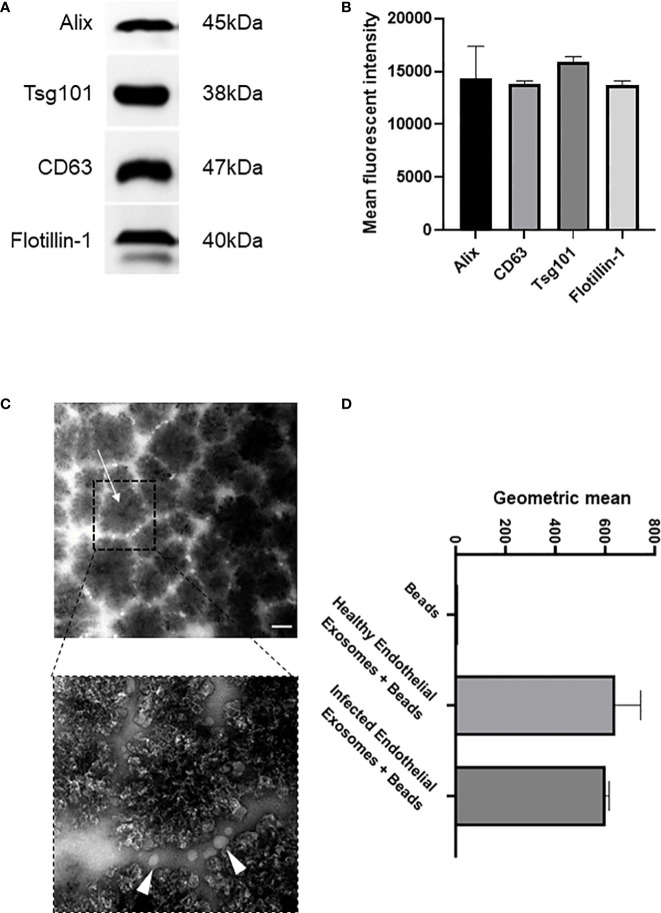
Isolation and characterisation of exosomes from healthy and infected endothelial cell supernatant **(A)**. Immunoblot of exosomal markers Alix (45 kDa), CD63 (38 kDa), Tsg101 (47 kDa), and Flotiliin-1 (40 kDa) isolated from the serum of *S. aureus* infected endothelial cells and healthy control. Exosomes were isolated by differential centrifugation and nanofiltration. **(B)** Densitometry representing western blot data of exosomal markers. **(C)** Transmission electron microscopy images represent superparamagnetic capture beads incubated with healthy endothelial exosomes and *S. aureus* infected endothelial exosomes. Images were taken at 50,000X (top) and 150,000X (bottom) magnification. Scale bar: 1 µm. Arrows point towards CD63 bead alone and CD63 bead bound to exosomes. **(D)** Flow cytometry analysis of CD63 beads bound to either healthy or infected endothelial exosomes. Data is representative of 3 independent experiments.

Exosomes are actively released from cells to facilitate intercellular communication in both healthy and disease states, and profiling exosomes can be particularly difficult due to their small size and low refractive index (Gardiner et al., 2014). However, exosomes commonly express various membrane and biogenesis related proteins which can distinguish subsets of extracellular vesicles. We confirmed by Western blot that our enriched samples did indeed contain exosomes since they expressed exosomal-specific markers including tetraspanin CD63, biogenesis related Alix and Tsg101, and membrane-associated Flotillin-1 (**Figures 1A, B**). Tsg101 was only detected in exosome samples and not microparticle fractions demonstrating that exosomal preparations were not contaminated with other vesicles or cellular components (**Supplementary Figures 1, 2**). In addition, we used an immunostaining approach wherein the enriched samples were analysed for their expression of CD63, a signature exosome surface marker (**Figure 1C**). Transmission electron microscopy was performed with 300 nm magnetic beads coated with the CD63 ligand. Images were acquired before and after incubation with exosomes purified from the endothelial cells, which demonstrated a collection of <100 nm sized vesicles that distributed around the beads (arrows). We next investigated if there was a difference in exosomal release from human endothelial cells following infection. Superparamagnetic CD63+ capture beads were used to detect exosomes from our enriched supernatants which were then analysed using flow cytometry. We found that there was no significant difference in exosomal release from human endothelial cells in healthy or infected conditions (**Figure 1D**, **Supplementary Figures 3, 4**).

### Analysis of exosomal miRNA derived from healthy and infected endothelial cells

**Figure 2:**
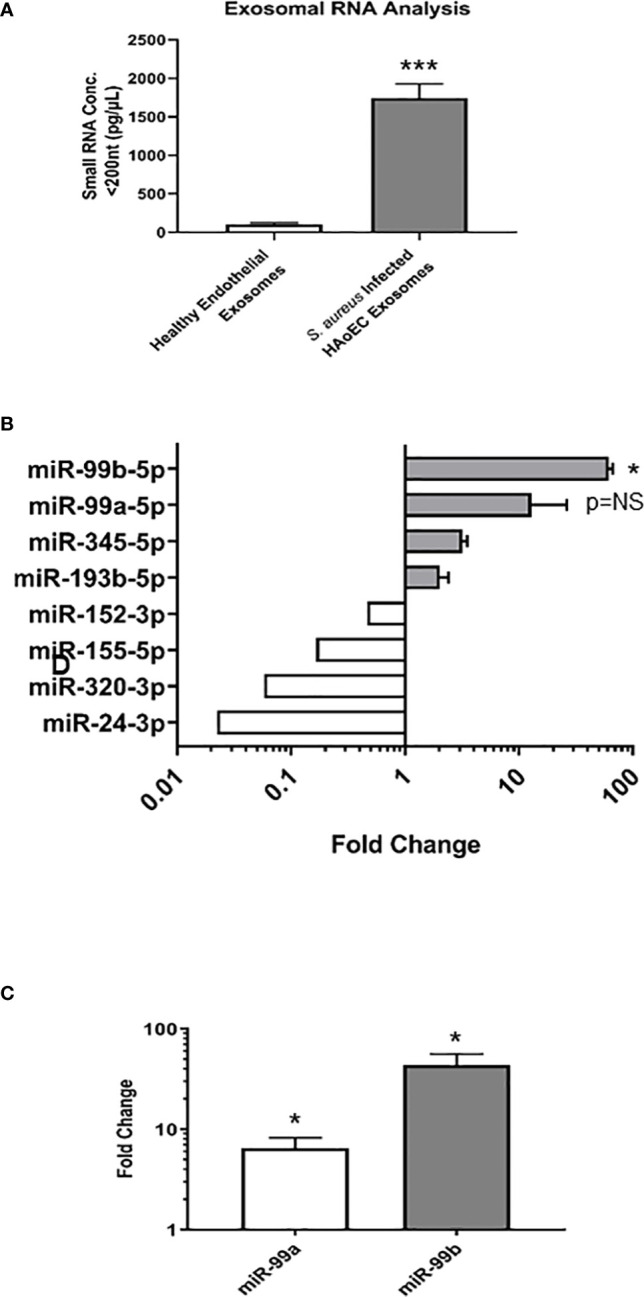
Characterisation of microRNA in healthy and infected endothelial cells **(A)**. Total RNA was extracted and isolated from healthy and *S. aureus* infected endothelial exosomes using Total Exosome RNA & Protein Isolation Kit and quantified using an AATI fragment analyser. This yielded a comprehensive miRNA expression profile for healthy and *S. aureus* infected endothelial exosomes. Individual miRNA expression data were calculated using the comparative cycle threshold (2-ΔΔCt) method, which indicated a significant increase in smRNA composition as a result of *S. aureus* infection **(B)** Differential expression of four upregulated and downregulated miRNA shown by fold change, with significantly high levels observed for miR-99b relative to healthy endothelial exosomes. **(C)** miR-99a and miR-99b levels in infected endothelial exosomes were validated using individual real-time PCR. Significant changes in both miRNAs were observed, with a 5.11-fold increase in miR-99a expression and a 38.82-fold increase in miR-99b expression. Data is representative of 4 independent experiments, as mean ± SEM. Statistics were performed using a two-tailed paired t-test, (*p<0.05, ***p<0.0001). NS, not significant.

Since similar levels of exosomes were found in both the healthy and infected endothelial cells, we next sought to characterise the exosomal contents in each condition to determine their miRNA expression profiles. Exosomes were isolated from healthy and *S. aureus* infected endothelial cells, then exosomal small RNA (smRNA) was quantified using a fragment analyser (**Figure 2A**). A 17-fold increase in smRNA was observed in exosomes isolated from the infected endothelial cells, in contrast to the exosomes from the healthy endothelial cells. Spectrometer RNA profiles indicated that exosomes isolated from healthy and infected cells contain smRNA between 4 and 40 nucleotides in length, consistent with miRNA. We performed quantitative miRNA expression analysis using TaqMan™ Array Human MicroRNA A Cards containing 384 miRNAs, to determine the miRNA profile of the exosomes derived from the healthy and infected endothelial cells (**Figure 2B**). Briefly, we detected the presence of 49 differentially regulated miRNAs altered in response to *S. aureus* infection, of which 17 were upregulated and 14 downregulated. In particular, the microRNA-99 family members (miR99a/b) were highly upregulated, both of which are putative downstream targets of mTOR – a protein associated with the anti-inflammatory response to microbial infection (Weichhart et al., 2011; Cao et al., 2017; Tsai et al., 2018). To validate exosomal miRNA expression, individual TaqMan arrays were performed which revealed that miR-99a and miR-99b were upregulated in contrast to exosomes derived from healthy endothelial cells (**Figure 2C**). Collectively, these results suggest that there are significant differences in the miRNA profile in exosomes isolated from healthy and *S. aureus*-infected human endothelial cells. Specifically, exosomal miRNA-99a/b may be associated with infection through the mTOR regulation pathway.

### Delivery of endothelial exosomes to monocytes and absolute determination of miR-99 concentration in monocytes

Monocytes are innate immune cells that play a central role in the host response to infection. They function to trigger an inflammatory response to eradicate the infectious pathogen, thereby are critical in modulating sepsis-induced inflammation and immunosuppression (Giamarellos-Bourboulis et al., 2006; Santos et al., 2016). In normal physiological conditions, endothelial cells are quiescent and do not interact with monocytes (Njock et al., 2015; Tang et al., 2016). In an inflammatory and infectious environment, vascular activation promotes the expression of surface adhesins which in turn binds monocytes (Tang et al., 2016). In addition, emerging evidence suggests under pathological conditions, exosomes may alter the contents of their cargo and deliver them to neighbouring immune cells to promote intercellular communication and microbial defence (de Jong et al., 2012; Wu et al., 2017; Barberis et al., 2021). Since our results revealed exosomal content released by endothelial cells is altered during *S. aureus* infection, we sought to determine whether endothelial-derived exosomes could be taken up by monocytes and whether this contributes to their immunomodulatory function in sepsis.

**Figure 3:**
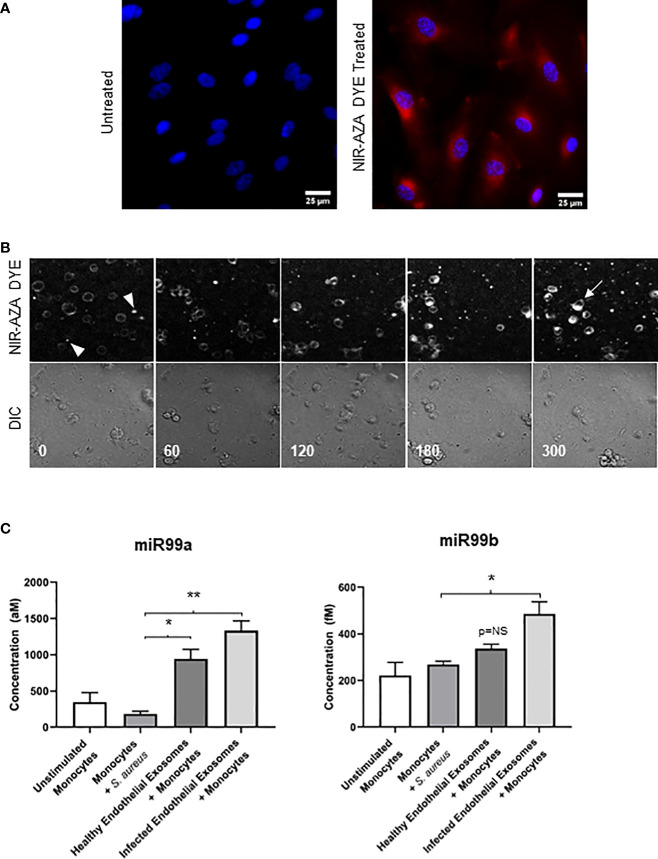
Delivering infected endothelial exosomes to monocytes causes increase in miR-99b and miR-99b levels **(A)** Endothelial cells were treated with Near-Infrared BF2-azadipyrromethene (NIR-AZA) dye (red) then viewed by fluorescent microscopy at 63X. Nuclei were counterstained with DAPI (blue). **(B)** Exosomes were isolated from cells and delivered to THP-1 monocytes *via* 8-channel ibidi chambers and analysed with real-time fluorescent video microscopy. Images were captured every 5 minutes over 16 hours. Images displayed using a time-lapse across 300 minutes (top panel: Cy5 channel, bottom panel: DIC channel). Absence of fluorescence at 0 minutes indicates monocytes have not yet interacted with exosomes (arrowheads). At 300 minutes, fluorescent intensity was greatest, and staining of the plasma membrane and cytosolic components of monocytes suggests exosomes were successfully taken up (arrow). **(C)** Real-time PCR using standard curves was performed to determine absolute quantification of miR-99a and miR-99b levels in monocytes following delivery of exosomes obtained from healthy and infected endothelial cells. No significant difference in miR-99a/b was noted when monocytes were directly infected with *S. aureus*. Delivery of healthy endothelial exosomes to monocytes induced a significant increase in miR-99a expression relative to unstimulated monocytes, respectively. Delivery of infected endothelial exosomes to monocytes induced a significantly higher increase in miR-99a and miR-99b levels. The cystolic increase in both miR-99 concentrations in monocytes indicates successful exosomal delivery and uptake. Data was representative of 3 independent experiments as mean ± SEM. Statistics were performed using one-way ANOVA (*p<0.05, **p<0.01). NS, not significant.

We first treated the endothelial cells with a Near Infrared BF2-azadipyrromethene (NIR-AZA) fluorescent probe which binds non-specifically to the plasma membrane and cytosol of cells (Gantier et al., 2012). Immunofluorescence analysis revealed the NIR-AZA probe was successfully internalised by endothelial cells (**Figure 3A**). Next, exosomes were isolated from NIR-AZA stained cells that were infected with *S. aureus* for 24 hours. To identify if monocytes were capable of taking up these extracellular vesicles, the stained endothelial exosomes were co-cultured with monocytes and real-time fluorescent microscopy was performed over 16 hours. At t=0 minutes, small fluorescent vesicles – the endothelial exosomes stained with the NIR-AZA probe – surround the unstained monocytes (**Figure 3B**). At 60 minutes, the monocytes become fluorescent as they begin to interact with the exosomes. The fluorescent intensity of the monocytes is greatest at 300 minutes, indicating that since the NIR-AZA dye has stained the plasma membrane and cytosol of the monocytes, they have successfully taken up the endothelial exosomes. To validate exosomal uptake by monocytes, we performed absolute quantification of miR-99a and miR-99b in monocytes that were co-cultured with the exosomes derived from healthy and infected endothelial cells. Real-time PCR revealed basal concentrations of miR-99a and miR-99b in monocytes were 400 aM and 205 fM, respectively. *S. aureus* infection alone had no impact on miR-99a/b expression in monocytes. Delivery of healthy endothelial exosomes to monocytes induced a significant increase from basal levels in miR-99a (P<0.05), but not for miR-99b. Alternatively, delivery of *S. aureus*-infected endothelial exosomes caused a significant increase in both miR-99a (P<0.01) and miR-99b (P<0.05) expression in monocytes (**Figure 3C**). The cystolic increase in miR-99 levels in monocytes following exosome delivery confirms their uptake, relative to the unstimulated monocytes.

### Inflammatory phenotypic analysis of monocytes following endothelial exosome delivery

**Figure 4:**
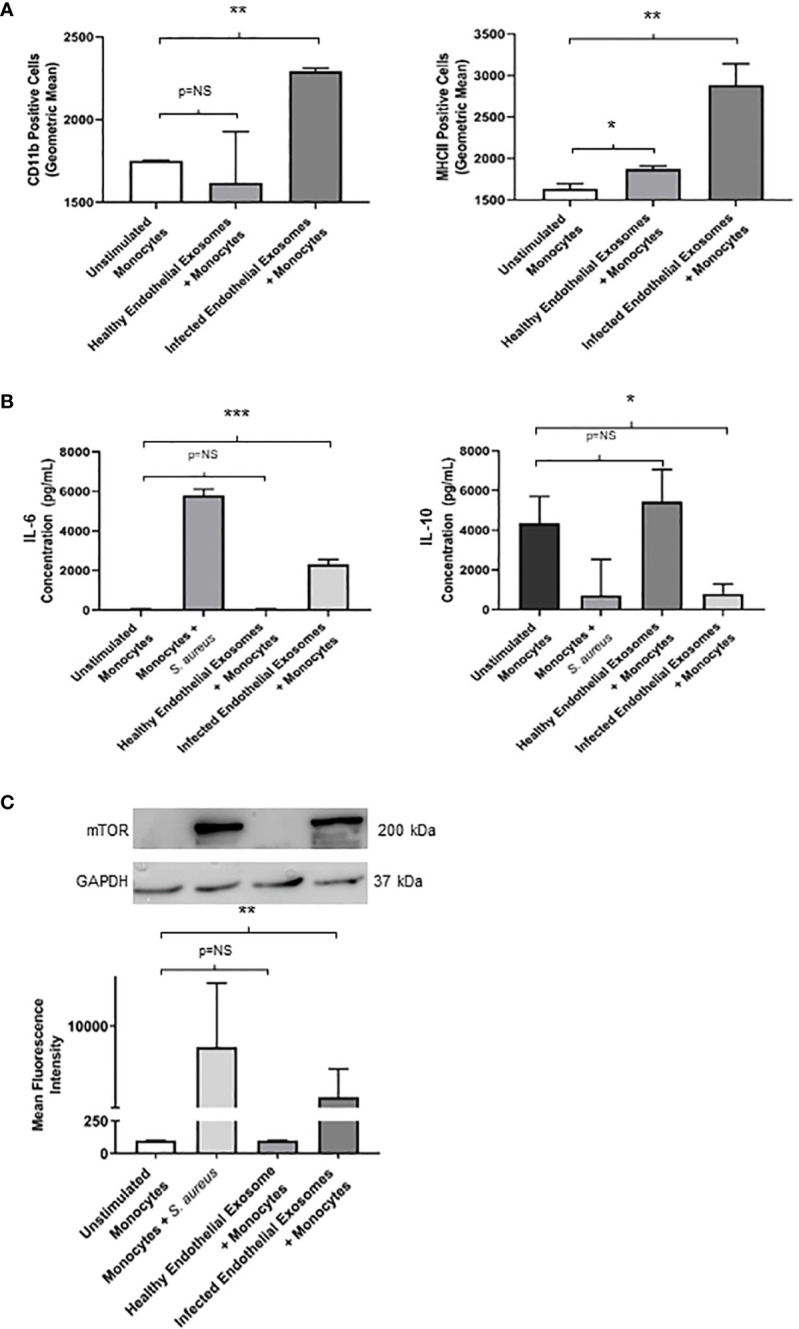
Infected endothelial exosomes promote a proinflammatory phenotype in monocytes following uptake **(A)** Expression of proinflammatory markers CD11b and MHCII were analysed on the surface of monocytes by flow cytometry after 24 hours. Data is represented as bar graphs using geometric mean taken from baseline negative control. Significant increases in CD11b and MHCII levels were observed in monocytes that had taken up infected exosomes. **(B)** Basal levels of IL-6 (35 pg/mL) and IL-10 (4300 pg/mL) were obtained in unstimulated THP-1 monocytes by ELISA. Direct *S. aureus* infection had the greatest influence on IL-6 and IL-10 production from monocytes. Healthy endothelial exosomes had no effect on cytokine expression compared to unstimulated monocytes. Exosomes from *S. aureus* infected endothelial cells induced a significant increase in IL-6 and a negatively correlating decrease in IL-10 production in monocytes. **(C)** Western blot and densitometry analyses of mTOR levels (200 kDa) in monocytes following delivery of healthy or infected exosomes after 24 hours. GAPDH (37 kDa) used as loading control. Positive control (monocytes + *S. aureus*) showed greatest mTOR protein expression. Significantly elevated mTOR levels were observed in monocytes following uptake of *S. aureus* infected endothelial exosomes. Data is represented as mean ± SEM (n=3 CD11b; n=4 MHCII; *p<0.05; **p<0.01, ***p<0.001). NS, not significant.

Having demonstrated successful uptake of endothelial exosomes by monocytes *in vitro* through real-time fluorescent microscopy and absolute quantification, we investigated possible pro-inflammatory effects induced by the exosomes. CD11b and MHCII are pro-inflammatory activation markers expressed on the surface of monocytes; CD11b is involved in monocyte adhesion, migration, and stimulation of pro-inflammatory pathways during infection, whereas MHCII is associated with antigen presentation and the adaptive immune response. Both proteins are routinely upregulated in response to a pro-inflammatory stimuli (Cella et al., 1997; Duan et al., 2016). Flow cytometry was performed to determine expression of CD11b and MHCII in monocytes following delivery of healthy and infected endothelial-derived exosomes after 24 hours (**Figure 4A**). A significant increase of both CD11b (P<0.05) and MHCII (P<0.05) expression was observed in the monocytes cultured with infected endothelial exosomes, compared to the unstimulated control group. Exosomes from healthy endothelial cells had no effect on CD11b but significantly increased MHCII expression. This suggests that *S. aureus* infected endothelial exosomes significantly alters CD11b and MHCII expression of monocytes which could directly influence their pro-inflammatory response to infection.

To further investigate the pro-inflammatory effect of exosomal delivery had on monocytes, we analysed the production of two major inflammatory cytokines frequently associated with sepsis mortality: IL-6 and IL-10. Basal levels of IL-6 and IL-10 were assessed in unstimulated monocytes. Healthy endothelial exosomes had no effect on cytokine expression whereas exosomes from *S. aureus* infected endothelial cells resulted in an increase in IL-6 (P<0.05) and a decrease in IL-10 (P<0.05) after 24 hours (**Figure 4B**). These results suggest that exosomes from *S. aureus* infected endothelial cells are driving the pro-inflammatory cytokine response and diminishing the anti-inflammatory response.

### mTOR activity is suppressed by miR-99a/b which promotes an anti-inflammatory effect during *S. aureus* infection

**Figure 5:**
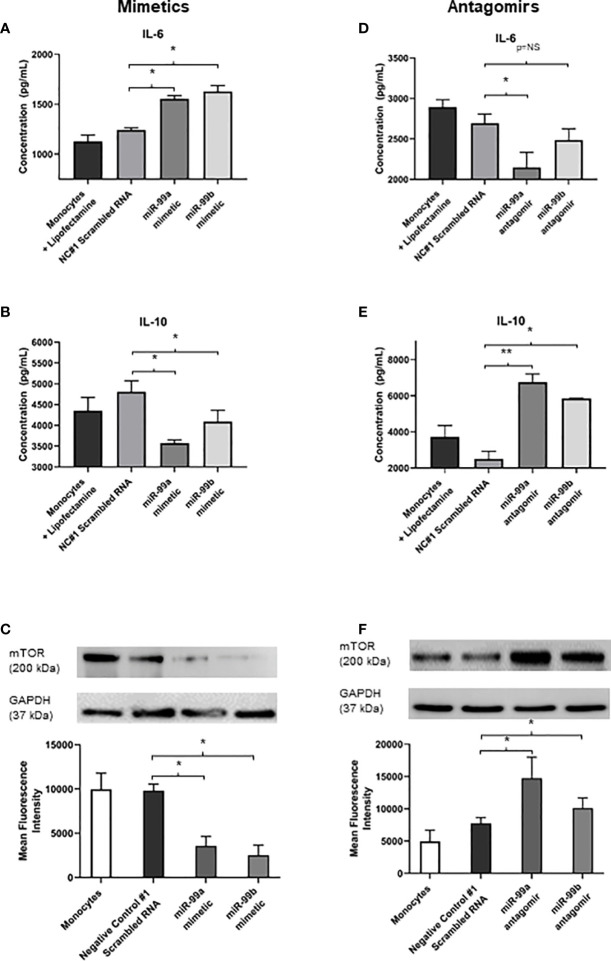
Exosomal miR-99a and miR-99b drives the proinflammatory response in monocytes by downregulating mTOR following *S. aureus* infection **(A, B)** IL-6 and IL-10 expression in monocytes treated with miR-99a/b mimetics following a 24 hour *S. aureus* infection was determined by ELISA. Monocytes treated with lipofectamine 3000, and negative control scrambled RNA were considered negative controls, where neither group showed significant differences in cytokine levels. Monocytes transfected with miR-99a and miR-99b mimetics resulted in a significant increase to IL-6 levels, which also observed a significant decrease in IL-10 expression. **(C)** Total mTOR expression in monocytes treated with miR-99a/b mimetics in the presence of a 24 hour *S. aureus* infection was determined by western blot and densitometry analyses. GAPDH was used as a loading control. Transfection of either the miR-99a or miR-99b mimetic caused a significant reduction in mTOR production. **(D, E)** MiR-99a and miR-99b antagomirs were transfected into monocytes in the presence of *S. aureus* infection. IL-6 and IL-10 levels were assessed using ELISA. miR-99a antagomirs resulted in significant decrease to IL-6 production yet miR-99b antagomirs maintained IL-6 at physiological levels compared to negative controls. **(D)** Knockdown of miR-99a and miR-99b using antagomirs resulted in a significant increase in IL-10 production relative to negative control scrambled RNA. **(E)**, **(F)** Total mTOR expression of monocytes treated with miR-99a/b antagomirs following a 24 hour *S. aureus* infection was determined by western blot and densitometry analyses. Both antagomirs resulted in a significant increase in mTOR levels relative to the negative controls. Comparatively, IL-6 and IL-10 production directly correlated with the changes in mTOR activity. Data is represented as mean ± SEM (n=6 western blot; n=4 ELISA; *p<0.05). NC, Negative control. **P<0.01, NS, not significant.

We identified that exosomes derived from endothelial cells infected with *S. aureus* contain heightened levels of miR-99, which are confirmed to target mTOR (Jin et al., 2013; Cao et al., 2017; Tsai et al., 2018; Yin et al., 2018). The mTOR pathway is a critical network which serves as master regulators of several cellular functions including metabolism, growth, and proliferation. In addition, mTOR limits the production of pro-inflammatory cytokines and promotes anti-inflammatory cytokines during infection (Weichhart et al., 2008; Weichhart et al., 2011). We performed western blots to assess mTOR protein expression in monocytes that were either unstimulated or co-cultured with exosomes derived from healthy and infected endothelial cells. Unstimulated and healthy endothelial exosomes produced undetectable mTOR levels, consistent with findings that mTOR activity is low in the absence of an appropriate stimuli (Zhang et al., 2011; Waickman and Powell, 2012; Jones and Pearce, 2017). In addition, monocytes directly infected with *S. aureus* caused heightened mTOR expression. However, monocytes exposed to infected endothelial exosomes had markedly lower mTOR expression than those infected with the bacteria directly (**Figure 4C**). This suggests that perhaps exosomal miR-99 expression could directly influence mTOR activity in monocytes during infection. To investigate this, transfection assays were performed using mimetics and antagomirs against miR-99a/b (**Figure 5A, D**). Mimetics targeting miR-99a, miR-99b, and a negative control composed of scrambled RNA were transfected into monocytes and subsequently infected with *S. aureus* for 24 hours. Both miR-99a and miR-99b mimetics delivered to monocytes resulted in reduced mTOR production (**Figure 5C**). We also analysed the effects of suppressing miR-99a/b using antagomirs (**Figure 5A**). Transfection of both miR-99a and miR-99b antagomirs to infected monocytes induced an increase in mTOR expression therefore restoring mTOR activity *in vitro* (**Figure 5F**). This data demonstrates that both members of the miR-99 family target and suppress mTOR activity during infection.

Since mTOR was successfully downregulated in the presence of miR-99a and miR-99b, we sought to investigate whether the production of pro- and anti-inflammatory cytokines could be influenced directly by these miRNAs during microbial infection. Basal levels of IL-6 and IL-10 were measured in negative controls with lipofectamine 3000 and scrambled RNA – both of which were infected with *S. aureus.* These were compared to *S. aureus* infected monocytes transfected with miR-99a and miR99b mimetics and antagomirs. Addition of miR-99a/b mimetics resulted in significantly increased levels of IL-6 and reduced IL-10 (**Figure 5B**); conversely, knockdown of miR-99a/b using antagomirs caused reduced IL-6 and increased IL-10 production (**Figure 5E**).

## Discussion

The intercellular signalling pathway that governs endothelial cross-talk during sepsis is determined by a complex interplay between pro- and anti-inflammatory mediators, and the nature of the sustained hyperinflammatory response that drives sepsis remains elusive. Here we demonstrate that human vascular endothelial cells may be responsible for the sustained hyperinflammatory response by utilising exosomes as signalling vehicles to modulate intercellular communication with immune cells.

Exosomes are important cargos which carry multiple mediators critical for the pathogenesis ofdisease such as carcinomas, neurodegenerative disorders i.e., Parkinson’s disease, and heart failure (Vella et al., 2008; Masyuk et al., 2013). A new body research now suggests that exosomes carry biological material such as DNA, proteins, mRNA, and miRNA capable of modulating the inflammatory response during infection (Barberis et al., 2021). Sepsis is described as a dysregulated host response to infection and associated organ dysfunction. Analysis of exosomes derived from septic mice revealed increased levels of pro-inflammatory cytokines including IL-1β, IL-2, IL-6, and TNF-α, in the early phase followed by IL-12, IL-15, IL-17, and IFN-γ, in the late phase (Gao et al., 2019). Additionally, human studies demonstrated that exosomes isolated from sepsis patients can induce endothelial cell caspase-3 activation and apoptosis by generating superoxide, NO and peroxynitrite (Gambim et al., 2007). Most interestingly, pharmacological inhibition of exosomes attenuated cytokine production in LPS treated mice (Essandoh et al., 2015). Expanding on these observations we found that exosomes specifically released from *S. aureus* infected human endothelial cells contain miRNA’s that have the potential to modulate transcriptional and translational programs within immune cells and therefore contribute to an excessive and sustained inflammatory response during sepsis.

Although a number of exosomal miRNAs have been identified in sepsis, the functional effect of these dysregulated miRNA’s has not yet been fully elucidated. Exosomal miR-155 and miR-19b have both been shown to be increased during sepsis (Alexander et al., 2015; Lv et al., 2020; Chen et al., 2021). Both of these miR’s induce the secretion of proinflammatory cytokines, including IL-6 and TNFα, by activating Nuclear Factor-κB (NF-ĸB) or through suppressor of cytokine signalling-1 (SOCS-1) (Gantier et al., 2012; Yang et al., 2015; Ye et al., 2016; Mann et al., 2018). In our study, we demonstrated that the total exosomal levels released from human endothelial cells do not differ between uninfected and infected conditions, however the contents of the exosomes were significantly different. We observed increased levels of miR-99a and miR-99b in infected endothelial derived exosomes. Both of these dysregulated miRNA’s specifically targeted mTOR, a key molecular switch in immune cells (Weichhart et al., 2011; Cao et al., 2017; Tsai et al., 2018). mTOR plays a crucial role in cell proliferation, migration, differentiation, and inflammation, and has been shown to limit the production of pro-inflammatory cytokines *in-vitro* following induction of cells with microbial stimuli (Weichhart et al., 2008; Dan et al., 2008; Zhou and Huang, 2011; Mori et al., 2014; Zeng and Chi, 2017).

During infection, mTOR maintains an anti-inflammatory environment through bidirectional regulation of NF-ĸB activity through IKKβ (Dhingra et al., 2013). Activation of mTOR results in dampening of anti-inflammatory cytokines and an increase in proinflammatory cytokines (Weichhart et al., 2008; Saemann et al., 2009; Weichhart et al., 2011). Monocytes are well characterised recipient cells for exogenous exosomes and play a key role in the sustained and excessive immune response during sepsis (Voorhees et al., 2013; Arango Duque and Descoteaux, 2014). Our data revealed that the exosomes derived from infected human endothelial cells interacted with monocytes to deliver miR-99a and miR-99b which induced a pro-inflammatory effect on the immune cells. The coordinated secretion of pro- and anti-inflammatory cytokines by monocytes is an essential defence mechanism during inflammation, yet the signalling pathways that contribute to this cytokine storm remain elusive (Nedeva et al., 2019). Monocytes directly infected with *S. aureus* experienced elevated mTOR, yet monocytes treated with infected endothelial exosomes had notably reduced mTOR levels. This could be due to high miR-99a/b within infected exosomes, since their overexpression from infected endothelial exosomes enhanced IL-6 production and dampened that of IL-10, whilst their antagomirs had the opposing effect. In addition, miR-99a/b mimetics suppressed mTOR expression significantly. Consistent with this, Weichert et al. demonstrated that inhibition of mTOR with rapamycin caused monocytes infected with the bacterium *Listeria monocytogenes* to have heightened pro-inflammatory effects, due to elevated cytokine production (IL-2, IL-6, TNF-α) (Weichhart et al., 2008; Weichhart et al., 2011).

Therefore, we proposed that mTOR is a key molecular switch that serves as an anti-inflammatory mediator and its inhibition enhances the pro-inflammatory state of cells. In addition, we have identified a novel mechanism by which endothelial exosomal miRNA drives this response. These findings suggest that miR-99a and miR-99b facilitates the pro-inflammatory behaviour of monocytes through mTOR suppression, and therefore could act as viable targets to dampen the hyperinflammatory response during sepsis. This analysis of endothelial exosomes following *S. aureus* infection has emphasized the crucial role that these nanovesicle intercellular communicators play in the pathophysiology of disease states.

## Data availability statement

The raw data supporting the conclusions of this article will be made available by the authors, without undue reservation.

## Ethics statement

The studies involving human participants were reviewed and approved by Royal College of Surgeons in Ireland Ethics Committee. The patients/participants provided their written informed consent to participate in this study.

## Author contributions

GF: Conceptualization, Formal analysis, Methodology, Manuscript preparation. DN: Formal analysis, Methodology, Manuscript preparation. RW: Methodology. CM: Resources, Analysis. GC: Conceptualization, Funding Acquisition, Analysis. SK: Conceptualization, Formal analysis, Funding acquisition, Supervision, Validation. All authors contributed to the article and approved the submitted version.

## Funding

The article was funded by the Irish Research Council under the grant GOIPG/2016/1094.

## Conflict of interest

The authors declare that the research was conducted in the absence of any commercial or financial relationships that could be construed as a potential conflict of interest.

## Publisher’s note

All claims expressed in this article are solely those of the authors and do not necessarily represent those of their affiliated organizations, or those of the publisher, the editors and the reviewers. Any product that may be evaluated in this article, or claim that may be made by its manufacturer, is not guaranteed or endorsed by the publisher.

## References

[B38] Alexander M. Hu R. Runtsch M. C. Kagele D. A. Mosbruger T. L. Tolmachova T.. (2015). Exosome-delivered microRNAs modulate the inflammatory response to endotoxin. Nat. Commun. 6, 7321. doi: 10.1038/ncomms8321 26084661 PMC4557301

[B52] Arango Duque G. Descoteaux A. (2014). Macrophage cytokines: involvement in immunity and infectious diseases. Front. Immunol. 5, 491. doi: 10.3389/fimmu.2014.00491 25339958 PMC4188125

[B23] Barberis E. Vanella V. V. Falasca M. Caneapero V. Cappellano G. Raineri D.. (2021). Circulating exosomes are strongly involved in SARS-CoV-2 infection. Front. Mol. Biosci. 8, 632290. doi: 10.3389/fmolb.2021.632290 33693030 PMC7937875

[B7] Buscaglia L. E. Li Y. (2011). Apoptosis and the target genes of microRNA-21. Chin. J. Cancer. 30 (6), 371–380. doi: 10.5732/cjc.30.0371 21627859 PMC3319771

[B17] Cao F. Liu T. Sun S. Feng S. (2017). The role of the miR-99b-5p/mTOR signaling pathway in neuroregeneration in mice following spinal cord injury. Mol. Med. Rep. 16 (6), 9355–9360. doi: 10.3892/mmr.2017.7816 29039596 PMC5779988

[B25] Cella M. Engering A. Pinet V. Pieters J. Lanzavecchia A. (1997). Inflammatory stimuli induce accumulation of MHC class II complexes on dendritic cells. Nature 388 (6644), 782–787. doi: 10.1038/42030 9285591

[B39] Chen M. Wang F. Xia H. Yao S. (2021). MicroRNA-155: Regulation of immune cells in sepsis. Mediators Inflamm. 2021, 8874854. doi: 10.1155/2021/8874854 33505221 PMC7810547

[B2] Chu M. Qin S. Wu R. Zhou X. Tang X. Zhang S.. (2016). Role of MiR-126a-3p in endothelial injury in endotoxic mice. Crit. Care Med. 44 (8), e639–ee50. doi: 10.1097/CCM.0000000000001629 26968021 PMC4949098

[B9] Cortez M. A. Bueso-Ramos C. Ferdin J. Lopez-Berestein G. Sood A. K. Calin G. A. (2011). MicroRNAs in body fluids–the mix of hormones and biomarkers. Nat. Rev. Clin. Oncol. 8 (8), 467–477. doi: 10.1038/nrclinonc.2011.76 21647195 PMC3423224

[B4] Cui M. Wang H. Yao X. Zhang D. Xie Y. Cui R.. (2019). Circulating micrornas in cancer: potential and challenge. Front. Genet. 10, 626. doi: 10.3389/fgene.2019.00626 31379918 PMC6656856

[B48] Dan H. C. Cooper M. J. Cogswell P. C. Duncan J. A. Ting J. P. Baldwin A. S. (2008). Akt-dependent regulation of NF-kB is controlled by mTOR and raptor in association with IKK. Genes Dev. 22 (11), 1490–1500. doi: 10.1101/gad.1662308 18519641 PMC2418585

[B24] de Jong O. G. Verhaar M. C. Chen Y. Vader P. Gremmels H. Posthuma G.. (2012). Cellular stress conditions are reflected in the protein and RNA content of endothelial cell-derived exosomes. J. Extracell Vesicles 1. doi: 10.3402/jev.v1i0.18396 PMC3760650 24009886

[B49] Dhingra R. Gang H. Wang Y. Biala A. K. Aviv Y. Margulets V.. (2013). Bidirectional regulation of nuclear factor-kappaB and mammalian target of rapamycin signaling functionally links Bnip3 gene repression and cell survival of ventricular myocytes. Circ. Heart Fail. 6 (2), 335–343. doi: 10.1161/CIRCHEARTFAILURE.112.000061 23395931

[B26] Duan M. Steinfort D. P. Smallwood D. Hew M. Chen W. Ernst M.. (2016). CD11b immunophenotyping identifies inflammatory profiles in the mouse and human lungs. Mucosal Immunol. 9 (2), 550–563. doi: 10.1038/mi.2015.84 26422753 PMC7101582

[B12] Duthie E. S. Lorenz L. L. (1952). Staphylococcal coagulase; mode of action and antigenicity. J. Gen. Microbiol. 6 (1-2), 95–107. doi: 10.1099/00221287-6-1-2-95 14927856

[B37] Essandoh K. Yang L. Wang X. Huang W. Qin D. Hao J.. (2015). Blockade of exosome generation with GW4869 dampens the sepsis-induced inflammation and cardiac dysfunction. Biochim. Biophys. Acta 1852 (11), 2362–2371. doi: 10.1016/j.bbadis.2015.08.010 26300484 PMC4581992

[B3] Felekkis K. Touvana E. Stefanou C. Deltas C. (2010). microRNAs: a newly described class of encoded molecules that play a role in health and disease. Hippokratia 14 (4), 236–240. 21311629 PMC3031315

[B36] Gambim M. H. do Carmo Ade O. Marti L. Verissimo-Filho S. Lopes L. R. Janiszewski M. (2007). Platelet-derived exosomes induce endothelial cell apoptosis through peroxynitrite generation: experimental evidence for a novel mechanism of septic vascular dysfunction. Crit. Care 11 (5), R107. doi: 10.1186/cc6133 17894858 PMC2556756

[B43] Gantier M. P. Stunden H. J. McCoy C. E. Behlke M. A. Wang D. Kaparakis-Liaskos M.. (2012). A miR-19 regulon that controls NF-kB signaling. Nucleic Acids Res. 40 (16), 8048–8058. doi: 10.1093/nar/gks521 22684508 PMC3439911

[B35] Gao K. Jin J. Huang C. Li J. Luo H. Li L.. (2019). Exosomes derived from septic mouse serum modulate immune responses *via* exosome-associated cytokines. Front. Immunol. 10, 1560. doi: 10.3389/fimmu.2019.01560 31354717 PMC6640201

[B14] Gardiner C. Shaw M. Hole P. Smith J. Tannetta D. Redman C. W.. (2014). Measurement of refractive index by nanoparticle tracking analysis reveals heterogeneity in extracellular vesicles. J. Extracell Vesicles. 3, 25361. doi: 10.3402/jev.v3.25361 25425324 PMC4247498

[B19] Giamarellos-Bourboulis E. J. Routsi C. Plachouras D. Markaki V. Raftogiannis M. Zervakis D.. (2006). Early apoptosis of blood monocytes in the septic host: is it a mechanism of protection in the event of septic shock? Crit. Care 10 (3), R76. doi: 10.1186/cc4921 16696867 PMC1550931

[B8] Hatziapostolou M. Polytarchou C. Iliopoulos D. (2013). miRNAs link metabolic reprogramming to oncogenesis. Trends Endocrinol. Metab. 24 (7), 361–373. doi: 10.1016/j.tem.2013.03.002 23602813

[B27] Jin Y. Tymen S. D. Chen D. Fang Z. J. Zhao Y. Dragas D.. (2013). MicroRNA-99 family targets AKT/mTOR signaling pathway in dermal wound healing. PloS One 8 (5), e64434. doi: 10.1371/journal.pone.0064434 23724047 PMC3665798

[B30] Jones R. G. Pearce E. J. (2017). MenTORing immunity: mtor signaling in the development and function of tissue-resident immune cells. Immunity 46 (5), 730–742. doi: 10.1016/j.immuni.2017.04.028 28514674 PMC5695239

[B13] Kerrigan S. W. Clarke N. Loughman A. Meade G. Foster T. J. Cox D. (2008). Molecular basis for staphylococcus aureus-mediated platelet aggregate formation under arterial shear in vitro. Arterioscler. Thromb. Vasc. Biol. 28 (2), 335–340. doi: 10.1161/ATVBAHA.107.152058 18063809

[B40] Lv L. L. Feng Y. Wu M. Wang B. Li Z. L. Zhong X.. (2020). Exosomal miRNA-19b-3p of tubular epithelial cells promotes M1 macrophage activation in kidney injury. Cell Death Differ. 27 (1), 210–226. doi: 10.1038/s41418-019-0349-y 31097789 PMC7206053

[B44] Mann M. Mehta A. Zhao J. L. Lee K. Marinov G. K. Garcia-Flores Y.. (2018). Author correction: An NF-kB-microRNA regulatory network tunes macrophage inflammatory responses. Nat. Commun. 9 (1), 3338. doi: 10.1038/s41467-018-05720-5 30115909 PMC6095848

[B33] Masyuk A. I. Masyuk T. V. Larusso N. F. (2013). Exosomes in the pathogenesis, diagnostics and therapeutics of liver diseases. J. Hepatol. 59 (3), 621–625. doi: 10.1016/j.jhep.2013.03.028 23557871 PMC3831338

[B45] Mori S. Nada S. Kimura H. Tajima S. Takahashi Y. Kitamura A.. (2014). The mTOR pathway controls cell proliferation by regulating the FoxO3a transcription factor *via* SGK1 kinase. PloS One 9 (2), e88891. doi: 10.1371/journal.pone.0088891 24558442 PMC3928304

[B53] Nedeva C. Menassa J. Puthalakath H. (2019). Sepsis: inflammation is a necessary evil. Front. Cell Dev. Biol. 7, 108. doi: 10.3389/fcell.2019.00108 31281814 PMC6596337

[B20] Njock M. S. Cheng H. S. Dang L. T. Nazari-Jahantigh M. Lau A. C. Boudreau E.. (2015). Endothelial cells suppress monocyte activation through secretion of extracellular vesicles containing antiinflammatory microRNAs. Blood 125 (20), 3202–3212. doi: 10.1182/blood-2014-11-611046 25838349 PMC4440888

[B1] Rudd K. E. Johnson S. C. Agesa K. M. Shackelford K. A. Tsoi D. Kievlan D. R.. (2020). Global, regional, and national sepsis incidence and mortality, 1990-2017: analysis for the global burden of disease study. Lancet 395 (10219), 200–211. doi: 10.1016/S0140-6736(19)32989-7 31954465 PMC6970225

[B50] Saemann M. D. Haidinger M. Hecking M. Horl W. H. Weichhart T. (2009). The multifunctional role of mTOR in innate immunity: implications for transplant immunity. Am. J. Transplant. 9 (12), 2655–2661. doi: 10.1111/j.1600-6143.2009.02832.x 19788500

[B5] Salvi V. Gianello V. Tiberio L. Sozzani S. Bosisio D. (2019). Cytokine targeting by mirnas in autoimmune diseases. Front. Immunol. 10, 15. doi: 10.3389/fimmu.2019.00015 30761124 PMC6361839

[B10] Samanta S. Rajasingh S. Drosos N. Zhou Z. Dawn B. Rajasingh J. (2018). Exosomes: new molecular targets of diseases. Acta Pharmacol. Sin. 39 (4), 501–513. doi: 10.1038/aps.2017.162 29219950 PMC5888687

[B18] Santos S. S. Carmo A. M. Brunialti M. K. Machado F. R. Azevedo L. C. Assuncao M.. (2016). Modulation of monocytes in septic patients: Preserved phagocytic activity, increased ROS and NO generation, and decreased production of inflammatory cytokines. Intensive Care Med. Exp. 4 (1), 5. doi: 10.1186/s40635-016-0078-1 26879814 PMC4754229

[B6] Soliman A. M. Das S. Abd Ghafar N. Teoh S. L. (2018). Role of microrna in proliferation phase of wound healing. Front. Genet. 9, 38. doi: 10.3389/fgene.2018.00038 29491883 PMC5817091

[B21] Tang N. Sun B. Gupta A. Rempel H. Pulliam L. (2016). Monocyte exosomes induce adhesion molecules and cytokines *via* activation of NF-kB in endothelial cells. FASEB J. 30 (9), 3097–3106. doi: 10.1096/fj.201600368RR 27226520 PMC5001509

[B11] Tilley D. O. Arman M. Smolenski A. Cox D. O’Donnell J. S. Douglas C. W.. (2013). Glycoprotein ibalpha and fcgammariia play key roles in platelet activation by the colonizing bacterium, streptococcus oralis. J. Thromb. Haemost. 11 (5), 941–950. doi: 10.1111/jth.12175 23413961

[B16] Tsai T. F. Lin J. F. Chou K. Y. Lin Y. C. Chen H. E. Hwang T. I. (2018). miR-99a-5p acts as tumor suppressor *via* targeting to mTOR and enhances RAD001-induced apoptosis in human urinary bladder urothelial carcinoma cells. Onco Targets Ther. 11, 239–252. doi: 10.2147/OTT.S114276 29379304 PMC5757495

[B34] Vella L. J. Sharples R. A. Nisbet R. M. Cappai R. Hill A. F. (2008). The role of exosomes in the processing of proteins associated with neurodegenerative diseases. Eur. Biophys. J. 37 (3), 323–332. doi: 10.1007/s00249-007-0246-z 18064447

[B51] Voorhees J. L. Tarr A. J. Wohleb E. S. Godbout J. P. Mo X. Sheridan J. F.. (2013). Prolonged restraint stress increases IL-6, reduces IL-10, and causes persistent depressive-like behavior that is reversed by recombinant IL-10. PloS One 8 (3), e58488. doi: 10.1371/journal.pone.0058488 23520517 PMC3592793

[B32] Waickman A. T. Powell J. D. (2012). mTOR, metabolism, and the regulation of T-cell differentiation and function. Immunol. Rev. 249 (1), 43–58. doi: 10.1111/j.1600-065X.2012.01152.x 22889214 PMC3419491

[B29] Weichhart T. Costantino G. Poglitsch M. Rosner M. Zeyda M. Stuhlmeier K. M.. (2008). The TSC-mTOR signaling pathway regulates the innate inflammatory response. Immunity 29 (4), 565–577. doi: 10.1016/j.immuni.2008.08.012 18848473

[B15] Weichhart T. Haidinger M. Katholnig K. Kopecky C. Poglitsch M. Lassnig C.. (2011). Inhibition of mTOR blocks the anti-inflammatory effects of glucocorticoids in myeloid immune cells. Blood 117 (16), 4273–4283. doi: 10.1182/blood-2010-09-310888 21368289

[B22] Wu J. Wang Y. Li L. (2017). Functional significance of exosomes applied in sepsis: A novel approach to therapy. Biochim. Biophys. Acta Mol. Basis Dis. 1863 (1), 292–297. doi: 10.1016/j.bbadis.2016.10.024 27989958

[B42] Yang Y. Yang L. Liang X. Zhu G. (2015). MicroRNA-155 promotes atherosclerosis inflammation *via* targeting SOCS1. Cell Physiol. Biochem. 36 (4), 1371–1381. doi: 10.1159/000430303 26159489

[B41] Ye J. Guo R. Shi Y. Qi F. Guo C. Yang L. (2016). miR-155 regulated inflammation response by the socs1-stat3-pdcd4 axis in atherogenesis. Mediators Inflamm. 2016, 8060182. doi: 10.1155/2016/8060182 27843203 PMC5098093

[B28] Yin H. Ma J. Chen L. Piao S. Zhang Y. Zhang S.. (2018). MiR-99a enhances the radiation sensitivity of non-small cell lung cancer by targeting mtor. Cell Physiol. Biochem. 46 (2), 471–481. doi: 10.1159/000488615 29614485

[B46] Zeng H. Chi H. (2017). mTOR signaling in the differentiation and function of regulatory and effector T cells. Curr. Opin. Immunol. 46, 103–111. doi: 10.1016/j.coi.2017.04.005 28535458 PMC5554750

[B31] Zhang S. Readinger J. A. DuBois W. Janka-Junttila M. Robinson R. Pruitt M.. (2011). Constitutive reductions in mTOR alter cell size, immune cell development, and antibody production. Blood 117 (4), 1228–1238. doi: 10.1182/blood-2010-05-287821 21079150 PMC3056471

[B47] Zhou H. Huang S. (2011). Role of mTOR signaling in tumor cell motility, invasion and metastasis. Curr. Protein Pept. Sci. 12 (1), 30–42. doi: 10.2174/138920311795659407 21190521 PMC3410744

