# Supplementary material for: Human endothelial cell-derived exosomal microRNA-99a/b drives a sustained inflammatory response during sepsis by inhibiting mTOR expression
Source: Front Cell Infect Microbiol. 2022 Aug 18;12:854126. doi: 10.3389/fcimb.2022.854126 (PMC9434345; doi:10.3389/fcimb.2022.854126)
Supplement: Supplementary file 1 [file DataSheet_1.docx]

**Supplementary material**

**
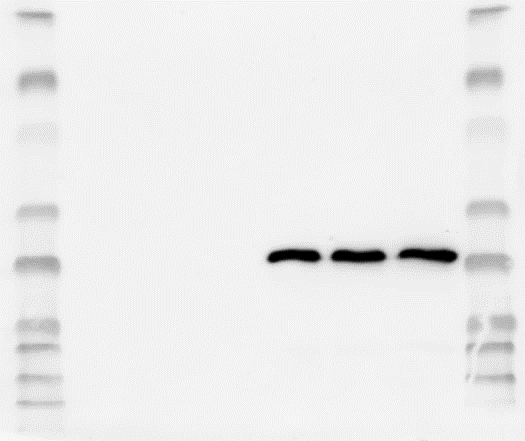

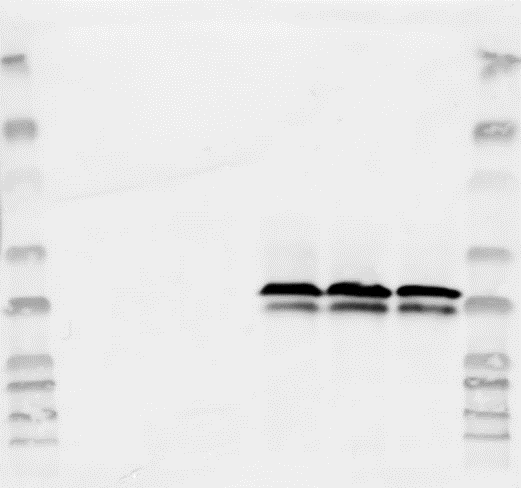

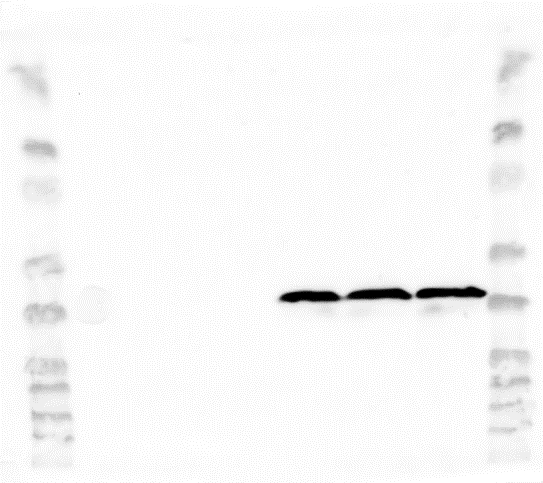

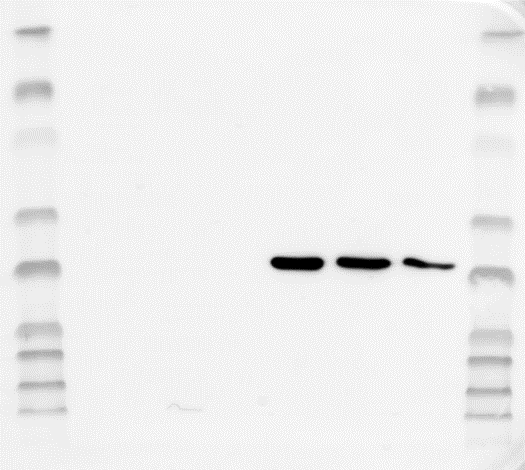
Figure 1:** Enrichment of exosomal markers including TSg101. Data was obtained from three independent experiments.

Flotillin-1

Tsg101

CD63

Alix

40kDa

47kDa

38kDa

45kDa

**Figure 2:** Differentiating exosomes from microparticles by Western blot. Microparticle fraction was obtained by resuspending pellets after 20,000 x g ultracentrifugation. Exosomes were subsequently isolated by passing the 20,000 x g supernatant through a 0.2µm filter and ultracentrifuging the filtrate at 120, 000 x g. Data was obtained from three independent experiments.


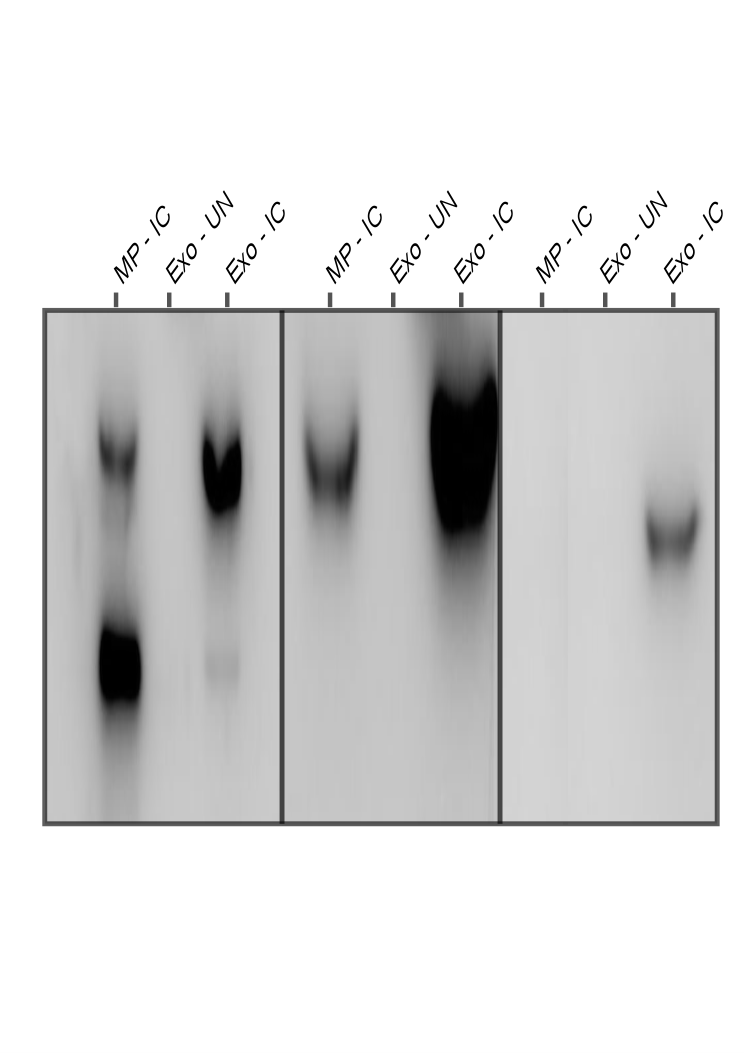

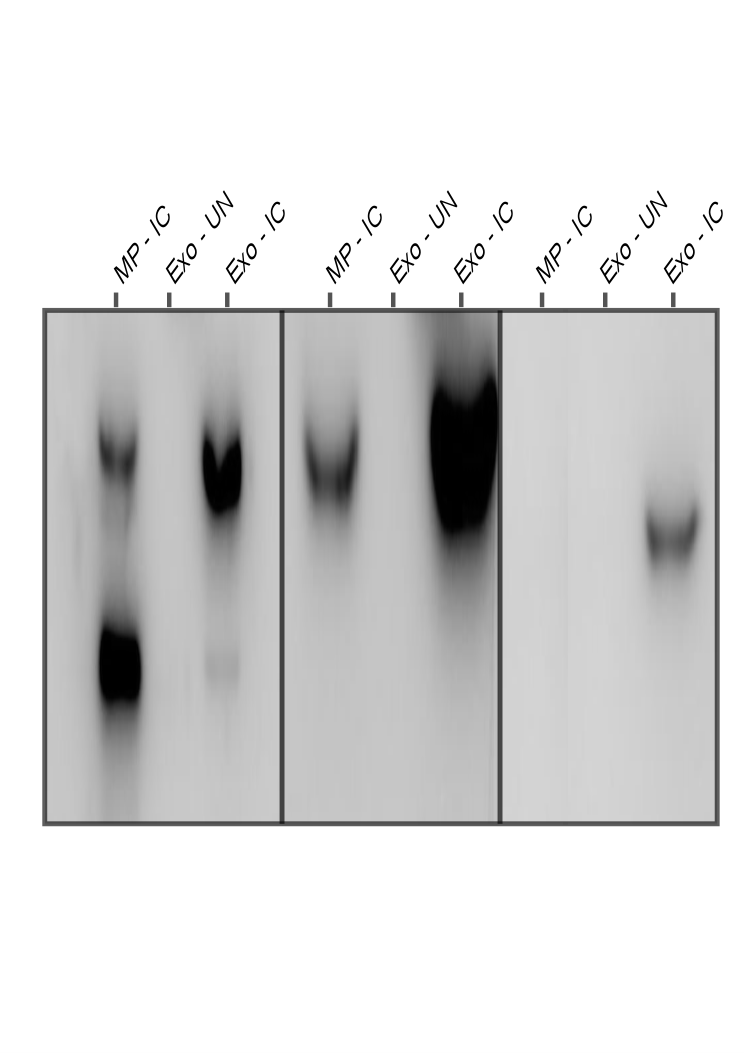

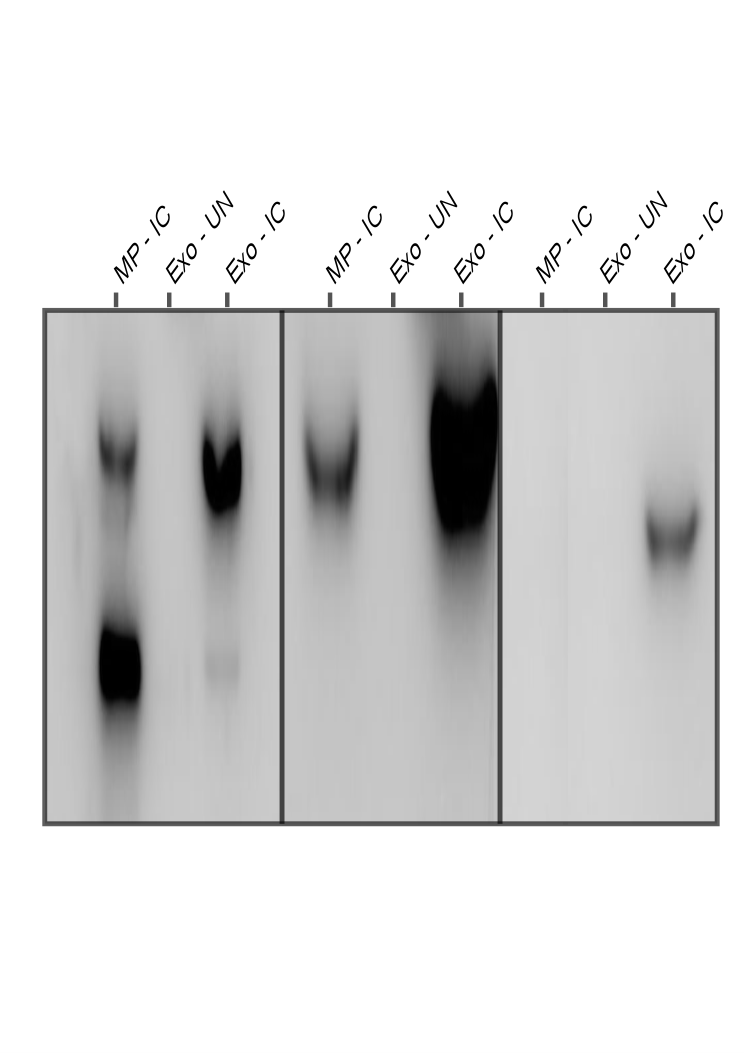


Exosomes

Microparticles

40kDa

Flotillin-1

38kDa

CD63

47kDa

Tsg101

**Figure 3:** Visualization of exosomes bound to CD63 capture beads by Transmission Electron Microscopy (TEM). A. Isolated superparamagnetic control beads alone placed on a PELCO 200 Mesh Special Metal Grid, at 150,000X. B. Superparamagnetic capture bead bound to exosomes taken at 50,000X (left) and 150,000X (right). White arrows point to bead, white arrowheads point to exosome.

**A**

CD63 Beads alone


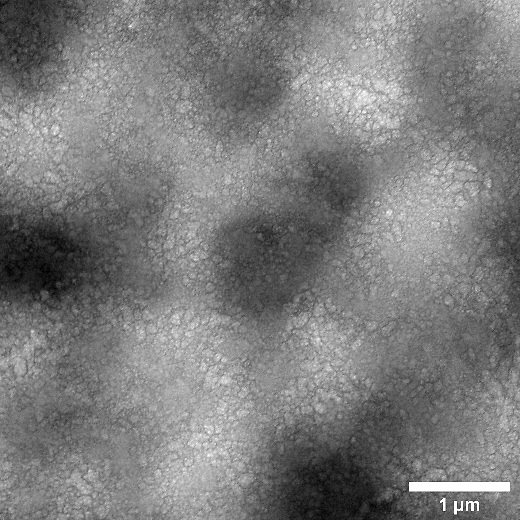

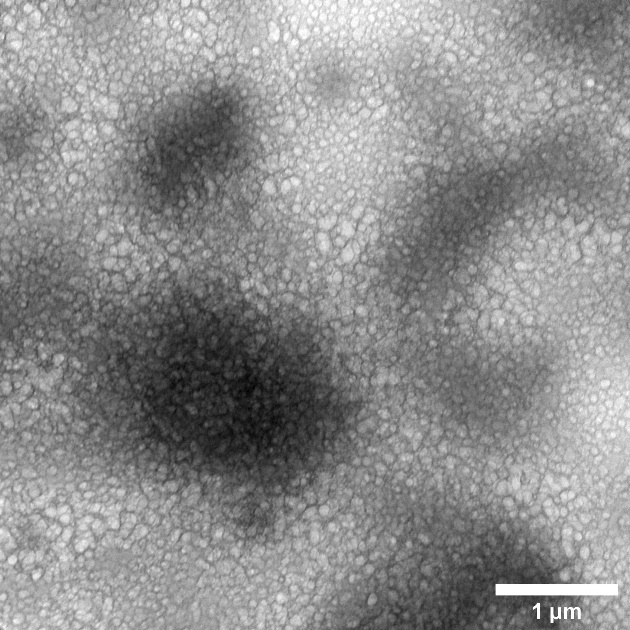

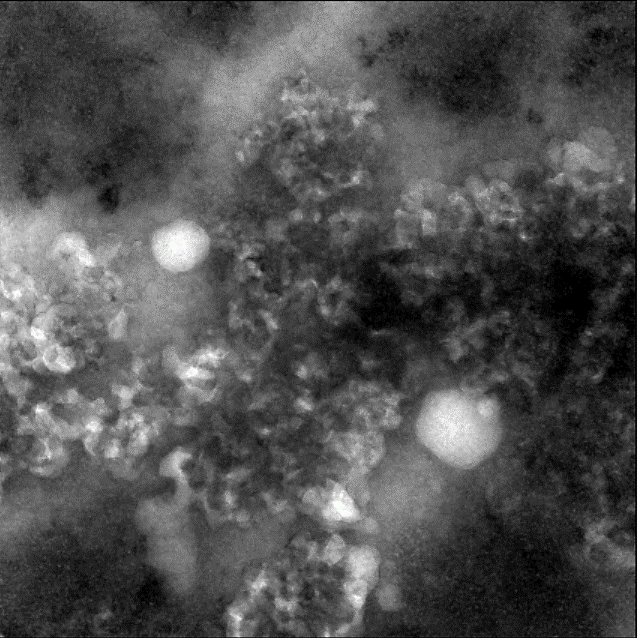


**B**

CD63 Beads + Exosomes


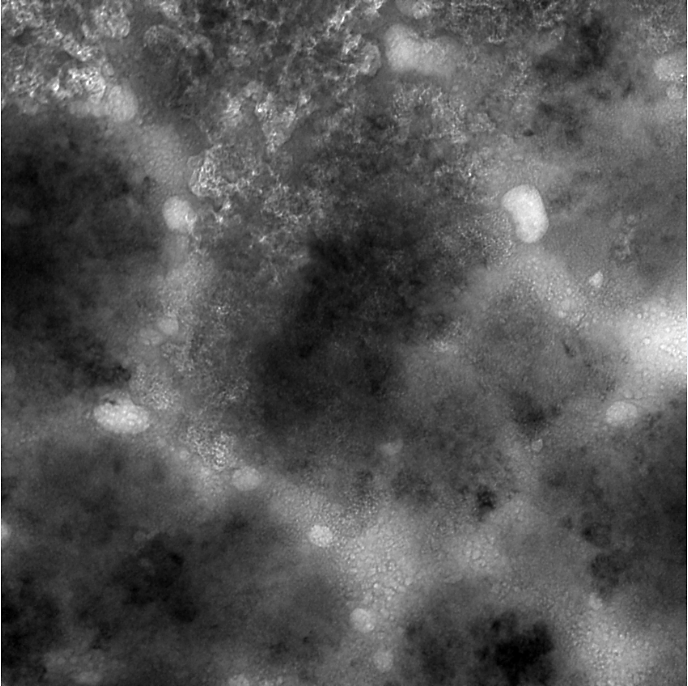


**Figure 4:** Isolation of CD63+ exosomes from supernatants of healthy and *S. aureus* infected endothelial cells by flow cytometry. Exosomes from were isolated by differential centrifugation and nanofiltration from cell culture supernatants of healthy and *S. aureus* infected endothelial cells and incubated with superparamagnetic capture beads containing anti-CD63 antibody (TEA3/18) A. Superparamagnetic capture beads incubated with endothelial growth media MV B. Superparamagnetic capture beads incubated healthy endothelial exosomes C. Superparamagnetic capture beads incubated *S. aureus* infected endothelial exosomes D. Compiled N=3 data (p=NS).

**D**

**B**

**B**

**A**

CD63 Beads + Healthy Endothelial Exosomes

CD63 Beads + Media


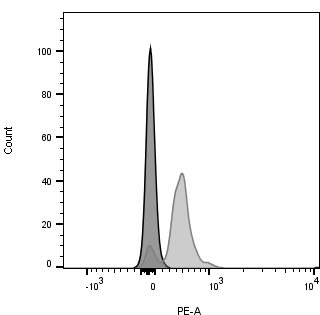

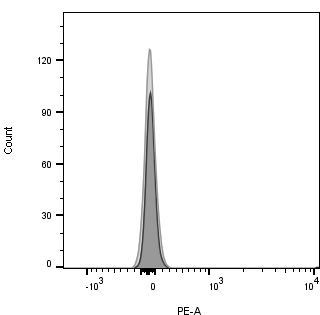

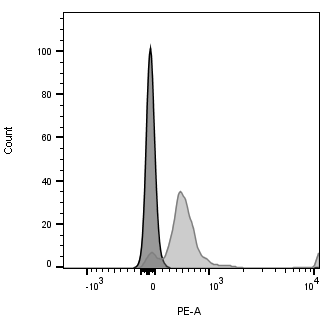

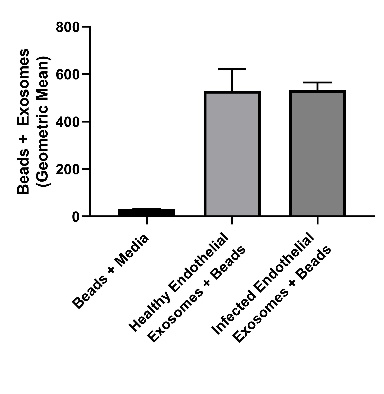


**D**

**C**

Count

Count

Count

PE-A

PE-A

PE-A

CD63 Beads + Infected

Endothelial Exosomes
